# Emotional State Recognition from Peripheral Physiological Signals Using Fused Nonlinear Features and Team-Collaboration Identification Strategy

**DOI:** 10.3390/e22050511

**Published:** 2020-04-30

**Authors:** Lizheng Pan, Zeming Yin, Shigang She, Aiguo Song

**Affiliations:** 1 School of Mechanical Engineering, Changzhou University, Changzhou 213164, China; 17000352@smail.cczu.edu.cn (Z.Y.); sheshg@cczu.edu.cn (S.S.); 2 Remote Measurement and Control Key Lab of Jiangsu Province, School of Instrument Science and Engineering, Southeast University, Nanjing 210096, China; a.g.song@seu.edu.cn

**Keywords:** emotion recognition, physiological signals, nonlinear features, identification strategy

## Abstract

Emotion recognition realizing human inner perception has a very important application prospect in human-computer interaction. In order to improve the accuracy of emotion recognition, a novel method combining fused nonlinear features and team-collaboration identification strategy was proposed for emotion recognition using physiological signals. Four nonlinear features, namely approximate entropy (ApEn), sample entropy (SaEn), fuzzy entropy (FuEn) and wavelet packet entropy (WpEn) are employed to reflect emotional states deeply with each type of physiological signal. Then the features of different physiological signals are fused to represent the emotional states from multiple perspectives. Each classifier has its own advantages and disadvantages. In order to make full use of the advantages of other classifiers and avoid the limitation of single classifier, the team-collaboration model is built and the team-collaboration decision-making mechanism is designed according to the proposed team-collaboration identification strategy which is based on the fusion of support vector machine (SVM), decision tree (DT) and extreme learning machine (ELM). Through analysis, SVM is selected as the main classifier with DT and ELM as auxiliary classifiers. According to the designed decision-making mechanism, the proposed team-collaboration identification strategy can effectively employ different classification methods to make decision based on the characteristics of the samples through SVM classification. For samples which are easy to be identified by SVM, SVM directly determines the identification results, whereas SVM-DT-ELM collaboratively determines the identification results, which can effectively utilize the characteristics of each classifier and improve the classification accuracy. The effectiveness and universality of the proposed method are verified by Augsburg database and database for emotion analysis using physiological (DEAP) signals. The experimental results uniformly indicated that the proposed method combining fused nonlinear features and team-collaboration identification strategy presents better performance than the existing methods.

## 1. Introduction

Emotions play an important role in human daily life. Currently, emotion recognition has received more and more attention in many fields, such as safe driving, distance education, health care and rehabilitation medical treatment [1]. Reliable and accurate emotion recognition system is one of the key problems of achieving natural human-machine interaction (HMI) [2].

In the last few decades, a variety of approaches for detecting human emotion have been performed by using speech, facial expression and behavior (gesture/posture) or physiological signals [3]. Leila et al. [4] proposed a global optimal feature fusion method for speech emotion recognition based on empirical mode decomposition and Teager-Kaiser Energy Operator (EMD-TKEO), according to the fact that the EMD combined with the TKEO gives an efficient time-frequency analysis of the non-stationary signals. In order to increase the accuracy rate of emotion recognition, unsupervised deep belief network (DBN) was proposed for depth level feature extraction from fused observations of Electro-Dermal Activity (EDA), Photoplethysmogram (PPG) and Zygomaticus Electromyography (zEMG) sensors signals and the fine Gaussian support vector machine was employed [5]. Hu et al. [6] proposed an integrated framework of two networks—a local network and a global network, which were based on local enhanced motion history image (LEMHI) and CNN-long short term memory (LSTM) cascaded networks respectively, for facial emotion recognition in video sequences. Picard et al. [7] proved that physiological signals can be used to classify emotions and successfully identified eight emotional states of calm, anger, disgust, sadness, peace, excitement, happiness and awe, using four physiological signals—electromyogram (EMG), pulse wave, skin conductivity (SC) and respiration (RSP). Many researchers have carried out researches on emotion recognition based on multi-modal physiological signals and EEG signals, many methods of emotion recognition were proposed, meanwhile, free emotion recognition databases with electroencephalogram (EEG) and peripheral neurophysiological signals were set up [8,9,10,11]. However, the informations of speech, facial expression and postures are more subjective [8]. Studies have shown that the signals controlled by the human nervous system [12,13,14,15,16,17,18], are closer to present people’s real emotions. Currently, using physiological signals for emotion recognition is one of the research hotspots.

In terms of using physiological signals to identify emotions, the core work of emotion identification mainly includes feature extraction and classification. In the aspect of feature extraction, numerous different features have been investigated for emotion recognition, from time domain, frequency domain, time-frequency domain and others. The extracted features based on the above mentioned analysis methods are considered inadequate when comes to use signals associated with the central nervous system (CNS) for emotion identification. On the one hand, a large number of features are needed to be extracted to represent the characteristics of each signal. On the other hand, the CNS related signals are non-stationary signals with characteristics varying over time and the traditional extraction methods can only achieve some superficial information [19,20,21]. However, the use of nonlinear analysis has the potential to provide discriminant features of CNS related signals that can enhance the classification accuracy of emotional states. In fact, nonlinear features have been successfully employed in a variety of ways. He et al. [22] employed wavelet packet entropy (WpEn) of speech signals as the feature to represent the emotion of angry, neutral and soft and achieved the accuracy of emotion recognition with 76%. Wanget al. [23] adopted three features, the approximate entropy (ApEn), hurst exponent and fractal dimension as extracted features from EEG and the performances of each feature with support vector machine (SVM) presented with 65.12% (ApEn), 71.38% (hurst exponent) and 70.63% (fractal dimension), respectively. Vayrynen et al [24] studied four type of emotional states (neutral, sad, angry and happy) with speech signals and employed two nonlinear features and k-nearest neighbour (KNN) classifier to realize emotion identification. In emotion identification, classifier also plays a very important role. The classifiers, SVM, DT, KNN and so on, are usually selected to realize emotion identification. Hosseini et al. [25] applied two entropy features and SVM to perform two types of emotion recognition with EEG. The sample entropy and SVM were adopted to realize two binary emotion recognition from EEG, with classification performances for positive and negative emotion by 80.43% and 79.11%, respectively [26]. Li et al. [27] adopted SVM to identify eight kinds of positive and negative emotions, combining the extracted features wavelet entropy (WaEn), ApEn and husrt exponent of EEG. Experimental results showed that the method achieved 85% recognition accuracy for two types of emotion recognition. Mohammadi et al [28] calculated the WaEn of each frequency band from EEG as the input feature of KNN classifier and achieved the classification accuracy of 86.75% for arousal level and 84.05% for valence level. According to the current research status, EEG signals are mainly selected for emotion recognition using signals related to the nervous system and the application of nonlinear features is relatively simple and not well integrated. In addition, when performing emotion recognition, a single classifier is usually used for recognition and determination.

As metioned above, physiological signals controlled by the human nervous system can also directly reflect emotional states. Because physiological signals are easy to obtain, this paper conducts research on emotion recognition from the perspective of using physiological signals. Previous researches of emotion recognition have largely been conducted on single-modality-based methods [12,17,24,25,26]. Compared with single-modality-based methods, fusing multiple sensors’ data is helpful to reflect emotional states from multiple perspectives, because the use of more than two modalities can exploit the complementary nature of different modalities so as to increase the credibility of measurements. In order to better represent the emotional state, this paper proposes the feature representation method of multi-nonlinear feature integration and multi-channel information feature fusion. Each classifier has its own performance characteristics. Samples that are misclassified by one classifier may easily be correctly classified by another classifier. In order to improve the accuracy of emotion identification, a team-collaboration dentification strategy is proposed, which can use different identification strategies to identify samples according to the characteristics of samples and the designed decision-making mechanism.

## 2. Materials and Methods

In this investigation, in order to effectively realize emotion identification, feature fusion of multi-channel signals is suggested to represent the emotional states and team-collaboration identification strategy is proposed to achieve emotion recognition. Two public databases, Augsburg dataset and DEAP dataset, are employed to verify the effectiveness and performances of the proposed methods.

### 2.1. Database

#### 2.1.1. Augsburg Dataset and Data Pre-Processing

The public emotional dataset [29] from University of Augsburg records the different emotional states caused by corresponding musics (https://www.informatik.uni-augsburg.de/en/chairs/hcm/projects/tools/aubt/). To induce the subject to unaffectedly feel four different emotions, joy, anger, sadness and pleasure, some music songs that the subject himself carefully handpicked were employed. The chosen songs can bring back some spectial memories to the subject in respect of targeted emotion classes. When the participant listened to music songs, four kinds of physiological signals were recorded, including electromyogram (ECG), electrocardiogram (EMG), respiration change (RSP) and skin conductivity (SC). The sampling frequency of the ECG was 256 Hz and the other three signals were 32 Hz. Each recording of physiological signal was chosen to be 120s in length. The experiment was performed for 25 days and 25 samples of each emotional state were obtained, totally 100 samples (four types of motional states). The content summary of Augsburg Database is shown in Table 1. In this investigation, each 120s signal was segmented into ten 12s-samples without overlapping. Finally, a total of 1000 samples were obtained.

#### 2.1.2. DEAP Dataset and Data Pre-Processing

Another publicly available database—DEAP [30] (http://www.eecs.qmul.ac.uk/mmv/datasets/deap/download.html)—was also adopted to investigate the universality of proposed methods. The DEAP dataset consists of 32 EEG channels and 8 peripheral physiological signals (electro-oculogram (EOG), galvanic skin response (GSR), blood volume pulse (BVP), RSP, EMG, skin temperature (SKT)) of 32 healthy subjects (labeled from s01 to s32) with half females and aged between 19 and 37. The EEG and physiological signals were recorded while each subject was watching 40 one-minute videos which were carefully selected to elicit different emotional states. In DEAP, each video clip was rated from 1 to 9 for arousal and valence by each subject after watching 40 trials of videos and the discrete rating value can be used as a classification label in emotion recognition. The total number of trials within the DEAP dataset was 1280 trials (40 trials per subject). 

In this research, four dimensional emotional states (high valence-high arousal (HVHA), high valence-low arousal (HVLA), low valence-low arousal (LVLA), low valence-high arousal (LVHA)) were considered as target emotions based on the scales of valence and arousal. Four kinds of peripheral physiological signals GSR, BVP, RSP, EMG from DEAP dataset were employed for emotion identification. The content summary of preprocessed DEAP database is shown in Table 2. The preprocessed signals in DEAP dataset were down-sampled to 128Hz and the length of each trial was 63 seconds, including 3s baseline signal and 60s watching recodings. In this paper, after removing the 3s baseline signal, each 60s trial was segmented into ten 6s samples without overlapping. Finally, each subject presented with a total of 400 (40 trials × 10 samples) samples and the samples of five subjects (s01, s02, s03, s04, s05) were chosen to verify the effectiveness of the proposed methods. The sample distribution is shown in Table 3. 

### 2.2. Emotion Labeling Schemes

**Figure 1:**
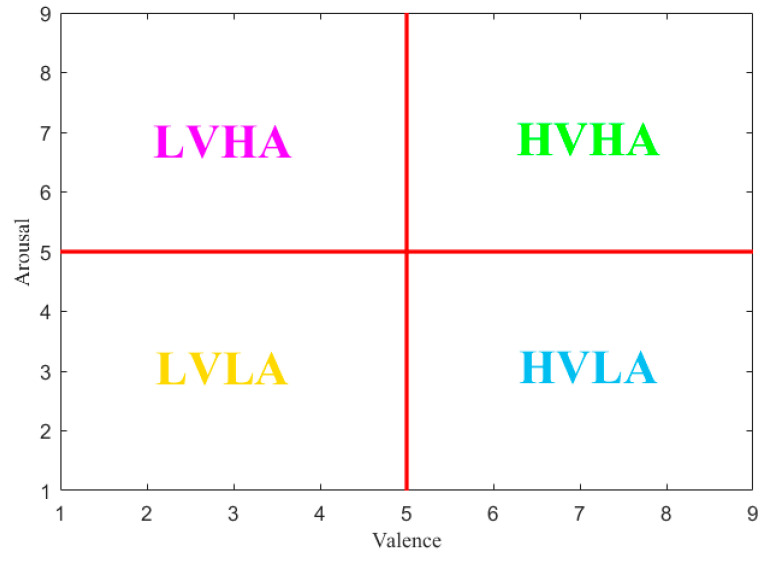
Emotion model classification based on the scale of valence and arousal.

In this research, two common types of emotion models were taken into consideration. One is to divide emotions into discrete categories. Ekman [31] regarded emotions as discrete and physiology related. He diveded emotions into six basic emotions with happy, sad, anger, fear, surprise and disgust and the other emotions were viewed as the production of reaction and combination of the six basic emotions. Izard [32] presented ten basic emotions—joy, sadness, angry, surprise, fear, interest, shyness, guilt, contempt and disgust. He suggested that each basic emotion corresponded to a simple brain circuit and there was no complex cognitive component involved. When using Augsburg Dataset, this discrete emotion model was employed to classify four different emotions, including joy, anger, sadness and pleasure, because the experimental emotional states were desigend with discrete categories. The other emotion model is based on the 2D arousal-valence emotion description model. In Russell’s theroy of emotion model [33], the emotional states are characterized by two dimension, valence and arousal. The emotion can be mapped to a plane with valence as the horizontal and arousal as the vertical axes. Arousal map emotions ranging from passtive to active while valence rangesfrom negative to positive. In this study, the DEAP dataset was divided into four-dimensional emotion groups according to Reference [30], including HVHA, HVLA, LVLA and LVHA based on the levels of valence and arousal, as shown in Figure 1. While valence > 5 and arousal > 5, it belongs to HVHA; while valence > 5 and arousal ≤ 5, it belongs toHVLA; while valence ≤ 5 and arousal > 5, it belongs to LVHA; while valence ≤ 5 and arousal ≤ 5, it belongs to LVLA. 

### 2.3. Feature Extraction

Prior to using the classifier for sample identification, features which can represent the emotional states usually need be extracted. The traditional linear analysis methods can only achieve some superficial information, therefore numerous features need be extracted from physiological signals to display the characteristics of signals, which reduces the efficiency of emotion classification. In addition, compared with periodic signals, emotion-related physiological signals are nonlinear time-varying signals. Therefore, in this investigation, four nonlinear features are employed to represent the emotional states from physiological signals. The four selected nonlinear features, approximate entropy (ApEn), sample entropy (SaEn), fuzzy entropy (FuEn) and wavelet packet entropy (WpEn), can reveal the internal structure of the time series and provide a prior knowledge about the intrinsic similarity, deep-seated information and predictability of the signal.

#### 2.3.1. Approximate Entropy 

Approximate entropy (ApEn) is a nonlinear analysis method proposed by S.M. Pincus [34,35,36] to measure the complexity or irregularity of time series. Its physical meaning is to measure the probability of mode generation in a new state when the signal sequence changes in its dimensional space. The method is based on the theory of phase space reconstruction. The core idea of this theory is that embedding the signal into the phase space, when the embedding dimension of the phase space is increased from *m* to *m + 1*, predicting the probability of generating the new mode. The greater the probability of generating a new mode, the more complex the signal and the higher the corresponding value of ApEn. The computed steps of ApEn are as follows:

(1) For time series $x_{i}$ of finite length *N* (1<i<N), $x_{i}$ is re-constructed into m-dimensional vector $X_{m}(i)$:

(1) $$\left\{ \begin{array}{l} X_{m}\left(i\right)=\left\{x\left(i\right),\;x\left(i+1\right),\;x\left(i+2\right),\;\ldots ,\;x\left(i+m-1\right)\right\}\\ i=1,\;2,\;\ldots ,N-m+1 \end{array}\right.,$$

where m is the embedded dimension.

(2) Calculate the distance $d\left\{X_{m}\left(i\right),X_{m}\left(j\right)\right\}$ between the components of $X_{m}\left(i\right)$ and $X_{m}\left(j\right)$ and define the maximum distance as $D\left\{X_{m}\left(i\right),X_{m}\left(j\right)\right\}$:

(2) $$D\left\{X_{m}\left(i\right),X_{m}\left(j\right)\right\}=max\left\{\left|x\left(i+k\right)-x\left(j+k\right)\right|\right\}.$$

(3) Calculate the probability of $C_{i}^{m}\left(r\right)$, which measures the regularity of $x_{i}$, that is, the ratio of $D\left\{X_{m}\left(i\right),X_{m}\left(j\right)\right\}<r$ to the total number of N−m+1:

(3) $$C_{i}^{m}\left(r\right)=\frac{N^{m}(i)}{N-m+1},$$

where r is the vector comparison distance and $N^{m}(i)$ represents the number of $D\left\{X_{m}\left(i\right),X_{m}\left(j\right)\right\}<r$.

(4) Define $\Phi^{m}\left(r\right)$ as:

(4) $$\Phi^{m}\left(r\right)=\frac{\sum_{i=1}^{N-m+1}\ln C_{i}^{m}(r)}{N-m+1}.$$

(5) When the dimension is increased to m+1, repeats the above steps to get $\Phi^{m+1}\left(r\right)$:

(5) $$\Phi^{m+1}\left(r\right)=\frac{\sum_{i=1}^{N-m}\ln C_{i}^{m+1}(r)}{N-m}.$$

(6) Finally, for fixed *m*, *r* and *N*, ApEn can be expressed as:

(6) $$ApEn(m,r,N)=\Phi^{m}\left(r\right)-\Phi^{m+1}\left(r\right).$$

The value of ApEn is related to vector comparison distance *r*, the embedded dimension *m* and the data length *N*. According to the practice of Pincus et al. [34], it is suggested that a relatively stable estimate can be obtained by using shorter data; *r* ranges from 0.1std to 0.25std, where *std* is the standard deviation of the data; the value of *m* is from 2 to 5.

#### 2.3.2. Sample Entropy 

Sample entropy (SaEn), proposed by Richman et al [37], is used to measure self-similarity and complexity of time series signals, being an improved algorithm based on ApEn. In view of the shortcomings of ApEn, SaEn mainly made two improvements—(a) Eliminating the deviation of ApEn when matching with itself; (b) In order to avoid the condition of ln0 caused by eliminating self-matching, SaEn calculates the total number of matching boards before logarithmic operation and when calculates *m + 1* dimensional statistics, the number of templates matching itself is used to calculate. The specific computing steps of SaEn are as follows:

(1) The first 3 steps are similar to the calculation of ApEn and define $Q_{i}^{m}\left(r\right)$ as the probability that $D\left\{X_{m}\left(i\right),X_{m}\left(j\right)\right\}<r$.

(2) After averaging all obtained $Q_{i}^{m}\left(r\right)$, gets the total number of template matches, $Q^{m}\left(r\right)$:

(7) $$Q^{m}\left(r\right)=\frac{\sum_{i=1}^{N-m}\ln Q_{i}^{m}(r)}{N-m}.$$

(3) When the dimension is increased to m+1, the total number of template matches is $Q^{m+1}\left(r\right)$:

(8) $$Q^{m+1}\left(r\right)=\frac{\sum_{i=1}^{N-m}\ln Q_{i}^{m+1}(r)}{N-m}.$$

(4) SaEn can be expressed as:

(9) $$SaEn(m,r,N)=-\ln\frac{Q^{m+1}\left(r\right)}{Q^{m}\left(r\right)}.$$

#### 2.3.3. Fuzzy Entropy 

Chen et al. [38,39] employed fuzzy theory to measure the complexity of signal sequence and proposed fuzzy entropy (FuEn) algorithm, which was successfully applied to the extraction and classification of EMG signals. Studies [40,41] show that FuEn algorithm has lower sensitivity and dependence on phase space dimension (*m*), similar tolerance limit (*r*) and the time series length (*N*) and contributes to the calculation efficiency. FuEn is computed as follows:

(1) The first 2 steps are similar to the calculation of ApEn. Define the maximum distance as $D_{ij}^{m}$:

(10) $$D_{ij}^{m}=max\left\{\left|x\left(i+k\right)-x\left(j+k\right)\right|\right\}.$$

(2) Imports fuzzy membership function $A_{ij}^{m}$:

(11) $$A_{ij}^{m}=\exp[-\ln(2)\cdot {\left(\frac{D_{ij}^{m}}{r}\right)}^{2}].$$

(3) Define $C_{i}^{m}\left(r\right)$ as:

(12) $$C_{i}^{m}\left(r\right)=\frac{\sum_{j=1,j\neq i}^{N-m+1}A_{ij}^{m}}{N-m}.$$

(4) Define $\Phi^{m}\left(r\right)$ as:

(13) $$\Phi^{m}\left(r\right)=\frac{\sum_{i=1}^{N-m+1}\ln C_{i}^{m}(r)}{N-m+1}.$$

(5) When the dimension is increased to *m + 1*, repeats the above steps and finally FuEn is:

(14) $$FuEn(m,r,N)=\ln\Phi^{m}\left(r\right)-\ln\Phi^{m+1}\left(r\right).$$

#### 2.3.4. Wavelet Packet Entropy 

Wavelet packet entropy (WpEn) is an algorithm that combines wavelet packet transform with information entropy, which takes the advantages of wavelet packet in accurately describing signals of different frequency bands and the information measurement of non-stationary signals based on information entropy. WpEn reflects the spectrum energy distribution of signals in different frequency band and can quantitatively describe the order or disorder degree of information distribution [42]. The specific algorithm steps of WpEn are as follows:

(1) The raw signal is decomposed into different signal components of different frequency bands by wavelet packet, the energy *E_i,j_* for each frequency range in each time window can be computed as:

(15) $$E_{i,j}(t)=\sum_{k=1}^{L_{i,j}}{\left(x_{i,k}\right)}^{2},$$

where *i* denotes the number of layers of wavelet packet decomposition; *j* denotes the *j*-th frequency band; *k* is the summation index; *L_i, j_* denotes the coefficient energy.

(2) Total energy $E_{total}$ of the signal in each time window is calculated as:

(16) $$E_{total}=\sum_{i=1}^{N}E_{i,j}.$$

(3) According to Shannon’s information entropy theory and the definition of wavelet packet energy, WpEn is defined as follows:

(17) $$WpEn=-\sum_{i}p_{i}\ln(p_{i}),$$

where $P_{i}=\frac{E_{i}}{E_{total}}$ is computed as the ratio between the energy of each level.

#### 2.3.5. Multimodal Feature Fusion

In this research, physiological signals are employed for emotion identification. The primary purpose of feature fusion is to improve the classification results by exploiting the complementary nature of different features [43,44,45]. Each of the four nonlinear features mentioned above can reflect the state characteristics of the signal to some extent from a certain point of view. Therefore, the early fusion of four nonlinear features combining as a single representation can reflect emotional states more effectively. Meanwhile, the feature fusion of multimodal physiological signals will be more comprehensive to represent emotional states from multiple perspectives. In this investigation, in order to represent emotional states effectively, we propose the feature representation method of multi-nonlinear feature integration and multi-channel information feature fusion. The four nonlinear features, are employed and extracted from every physiological signal, then the extracted features of various physiological signals are fused in order to represent the emotional states more effectively. 

### 2.4. Team-Collaboration Identification Strategy Based on SVM-DT-ELM

Some traditional classifiers, such as support vector machine (SVM), extreme learning machine (ELM) and decision tree (DT), have been well applied in emotion recognition, while each classifier has his own shortcomings inevitably. For instance, SVM has better diagnostic performance under small sample conditions, poor performance for large sample conditions and emerge multiple categories with the same number of votes when voting. The initial input parameters of ELM are generated randomly, which requires a large number of training samples and cannot guarantee the optimal parameters. DT is inconsistent with the data of different samples and the information gain tend to those features with more values. In view of the requirements for accuracy and reliability of emotion recognition system and the uncertainty caused by a single classifier, a team-collaboration identification strategy based on the fusion of SVM, DT and ELM, is proposed, which exerts the function of collaborative diagnosis with multiple classifiers, thus eliminating the uncertainty brought by a single classifier and improving the recognition accuracy. 

#### 2.4.1. Support Vector Machine 

Support vector machine (SVM) is a machine learning method based on the principle of structural risk minimization in statistical learning theory, which seeks the best performance between model complexity and learning ability to achieve the best generalization ability based on limited sample information [46,47,48]. The core idea is to realize nonlinear classification or regression fitting by mapping nonlinear classification or regression fitting problems into high-dimensional space by using kernel function to obtain the better classification or regression result. When making a decision in a classification problem, the voting method is usually adopted and the category with the most votes is the class to which the sample belongs.

For a data set $\left\{\left(x_{1},y_{1}\right),\left(x_{2},y_{2}\right),\;\ldots ,\;\left(x_{k},y_{k}\right)\right\}$ of two classes with *k* as the number of samples, where $x_{i}\in R^{n}$ represents the sample; $y_{i}\in \left\{+1,-1\right\}$ is the class label;  i=1,2,…,k is the training sample number. SVM seeks an optimal hyper-plane in the *n* dimensional data feature space by constructing the following function:

(18) $$\left\{ \begin{array}{l} \min\frac{1}{2}w^{T}w+C\sum_{i=1}^{n}\xi_{i}\\ y_{i}(w^{T}\phi (x_{i})+b)\geq 1-\xi_{i}\\ \xi_{i}\geq 0,i=1,2,3,\ldots ,n \end{array}\right.,$$

where ϕ is a mapping function from low-dimensional space to high-dimensional space; $\xi_{i}$ is a slack variable to ensure the correctness of the classification in the case of inseparable samples; *C* is a penalty factor and a larger *C* indicates a greater penalty for misclassification; *w* and *b* are the weight vector and classification threshold of the decision function f(x)=(w·x)+b; *x_i_* is the input vector and *y_i_* is the output vector.

Introducing the Lagrange function to get the dual optimization problem:

(19) $$\left\{ \begin{array}{l} \min\frac{1}{2}\alpha^{T}Q\alpha -e^{T}\alpha \\ y^{T}\alpha =0\\ 0\leq \alpha_{i}\leq C,i=1,2,3,\ldots ,n \end{array}\right.,$$

where $e={\left(1,1,\ldots ,1\right)}^{T}$ is the column vector; Q is the semi-positive definite matrix of n×n; $Q_{ij}=y_{i}y_{j}K(x_{i},x_{j}),K(x_{i},x_{j})=\phi {\left(x_{i}\right)}^{T}\phi {\left(x_{j}\right)}^{T}$ is the kernel function; $\alpha_{i}$ is the Lagrange multiplier; y is the sample label vector; α is the Lagrange multiplier vector.

Computing Equation (19), the optimal solution is:

(20) $$w=\sum_{i=1}^{n}y_{i}\alpha_{i}\phi (x_{i}).$$

The optimal hyper-plane decision function is:

(21) $$f(x)=\mathrm{sgn}(\sum_{i=1}^{n}y_{i}\alpha_{i}K(x_{i},x)+b).$$

SVM can be extended to multi-classification problems by constructing multiple SVM two-class classifiers which include direct method, one-to-one and one-to-rest. Among them, the one-to-one method is used to classify the *k* classes of sample data by constructing *k(k* *–1)/2* binary classifiers, which has a fast solving speed and is widely used in practice. The classification principle is the “voting mechanism,” that is, each classifier votes for its preference and the final result is based on the category with the most votes. This method can be expressed as:

(22) $$\left\{ \begin{array}{l} \min\left[\frac{1}{2}{\left(x^{ij}\right)}^{T}w^{ij}+C\sum_{i=1}^{n}\xi_{t}^{ij}\right]\\ {\left(w^{ij}\right)}^{T}\phi (x_{i})+b^{ij}\geq 1-\xi_{t}^{ij}(y_{t}=i)\\ {\left(w^{ij}\right)}^{T}\phi (x_{i})+b^{ij}\leq -1+\xi_{t}^{ij}(y_{t}=j)\\ \xi_{t}^{ij}\geq 0 \end{array}\right.,$$

where $w^{ij}$ and $b^{ij}$ are the weight vector and threshold obtained when designing the two-class classifier for the *i*-th sample and the *j*-th sample respectively; $\xi_{t}^{ij}$ is the slack variable; $x^{ij}$ is the training sample vector; $y_{t}$ is the sample label; *S* is the sum of the *i*-th class samples and *j*-th class samples.

#### 2.4.2. Decision Tree 

**Figure 2:**
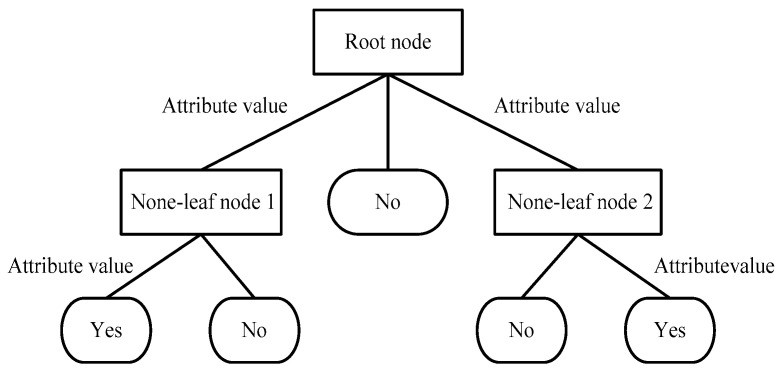
The structure diagram of decision tree (DT).

Decision tree (DT) classifier is an instance-based inductive learning algorithm that uses inductive algorithm to generate readable decision trees and rules and then uses the decision tree to classify new data [49]. DT is an inverted tree structure similar to the flow chart, which mainly focuses on the two core problems of growth and pruning. The structure diagram of DT is shown in Figure 2. The knowledge acquired by DT is a formal representation of the tree, including the regression tree and the classification tree. The results of classification or prediction are reflected in the leaf nodes of DT. The average value of the output variables is the prediction result in the samples contained in the leaf nodes of the regression tree, while the mode of the output variable is the classification result in the samples contained in the leaf nodes of the classification tree.

Each none-leaf node in the figure represents the input attribute of the training data set, attribute value represents the value corresponding to the attribute and the leaf node means the value of the target category attribute. Yes and No represent positive and negative examples respectively.

DT classifier is computed as follows:

Input—training set *D*, feature set *A* and threshold ε;

(1) If all samples in *D* belong to the same category of *C_k_*, then *T* is a single node tree and *C_k_* is used as the class of the node and returns *T*.

(2) If *A* is not an empty set, then *T* is a single node tree and the class *C_k_* with the largest number in *D* is taken as the class of the node and returns *T*.

(3) Otherwise, calculate the information gain ratio (GA) of each feature in *A* according to Equation (23) and select feature *A_g_* with the largest:

(23) $$\left\{ \begin{array}{l} GR=\frac{G}{SI}\\ G=Entropy(S)=\sum_{v\in V(A)}\frac{\left|S_{v}\right|}{\left|S\right|}Entropy(S_{v})\\ SI=\sum_{i=1}^{c}\frac{\left|S_{i}\right|}{\left|S\right|}\log2\frac{S_{i}}{S}\\ Entropy(S)=\sum_{i=1}^{c}-p_{i}\log2p_{i} \end{array}\right.,$$

where $p_{i}$ is the proportion of the sample of the *i*-th attribute value in the subset; *V(A)* is the range of the attribute *A*; *S_V_* is the subset of *D* whose value is *V* on the attribute *A*; Entropy(S) is the entropy of *D* relative to *C* states.

(4) If *A_g_* is less than ε, then *T* is a single node tree and the class *C_k_* with the largest number in *D* is taken as the class of the node and *T* is returned;

(5) Otherwise, for each possible value *a_i_* of *A_g_*, divide *D* into several non-empty subsets *D_i_* according to *A_g_ = a_i_*, mark the class with the largest number in *D_i_* as a mark to construct sub nodes and form a tree *T* by the node and return *T*.

(6) For node *i*, *D_i_* is used as training set and *A-{Ag}* as feature set. Step (1) to (5) are called recursively to get subtree *T_i_* and return *T_i_*.

#### 2.4.3. Extreme Learning Machine 

**Figure 3:**
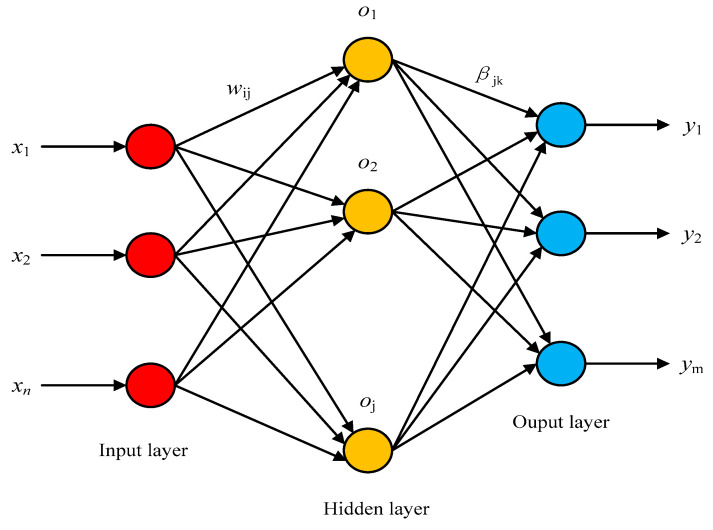
The network structure of Extreme Learning Machine (ELM).

In order to improve the traditional learning algorithms (such as back propagation neural network), which easily fall into local minimum, slow model training speed and difficulty in adjusting learning rate, reference [50] proposed the Extreme Learning Machine (ELM), which consisted of only an input layer, a hidden layer and an output layer. The brief network structure of the algorithm is shown in Figure 3. According the inputs, ELM randomly generates the connection weights and the threshold of hidden layer neurons between input layer and hidden layer, which need not be adjusted during the training process. Users do not need to know the hidden layer because Gaussian kernel was applied. The optimal solution can be obtained by setting the number of hidden layer neurons, which is related to the number of features. Best values for positive regularization coefficient and Gaussian kernel parameter were found empirically after several experiments.

Suppose there are *N* training samples $\left(x_{i},y_{i}\right)\in R^{N}\times R^{m}\left(i=1,\;2,\;\ldots ,\;N\right)$, where $x_{i}\in R^{N}$ is the input and $y_{i}\in R^{m}$ the output. The mathematical model of a standard single hidden layer feed forward neural network with *M* hidden layer nodes is:

(24) $$\sum_{i=1}^{M}\beta_{i}g(\omega_{j}x_{i}+b_{j})=o_{i},i=1,2,3,\ldots ,N,$$

where $\omega_{j}$ is the input weight vector connecting the input neuron and the *j*-th hidden layer neuron; $\beta_{j}$ is the output weight vector connecting the *j*-th hidden layer neuron and the output neuron; $o_{i}$ is the actual output vector; $b_{j}$ is the bias of the hidden layer neurons; g(x) is the activation function of the hidden layer neurons.

If the model can approximate the output $y_{j}$ of the training sample with zero error, which means $\sum_{i=1}^{N}o_{i}-y_{i}=0$, then $\beta_{j}$, $\omega_{j}$ and $b_{j}$ make the following formula hold:

(25) $$\sum_{i=1}^{M}\beta_{i}g(\omega_{j}x_{i}+b_{j})=y_{i},i=1,2,3,\ldots ,N.$$

Simplifies (25):

(26) Hβ=Y,

where H is called the output matrix of the hidden layer of the neural network.

When the activation function of the neuron is arbitrarily differentiable, the training error of the single hidden layer feed forward neural network can approach infinitely small positive number ε. At this time, the input weight vector $\omega_{j}$ and the hidden layer offset $b_{j}$ can remain unchanged during training process and can also be randomly assigned. Therefore, the training process is equivalent to finding the least squares solution $\hat{\beta }$ of the linear system:

(27) $$H\hat{\beta }-T=\underset{\beta }{\min}H\beta -T.$$

The solution is $\hat{\beta }=H^{+}Y$ and $H^{+}$ is the Moore-Penrose generalized inverse of the hidden layer output matrix H.

#### 2.4.4. Team-Collaboration Identification Strategy

As mentioned above, each classifier is based on a different method principle, so each classifier has its own advantages and disadvantages. For a sample, it may be easily misclassified by one classifier but easily identified by other classifiers. In order to reduce the limitation of a single classifier and improve the accuracy of recognition, a team-collaboration identification strategy model, which combines SVM, DT and ELM, is proposed. For this proposed team-collaboration identification strategy, the SVM model is regarded as a major decision expert and the DT and ELM are emloyed to provide the decision suggestions for the samples which are easily misclassified by SVM. The core idea of SVM-DT-ELM is that selecting the possibly misclassified samples for the SVM and then employing DT and ELM to conduct referral for these samples and finally confirming the emotion class of the sample according to the designed decision-making mchanism. Main procedures of the suggested SVM-DT-ELM algorithm are as follows:

(1) Firstly, the training sets are used to train SVM, DT, ELM classification model respectively. In this research, during training SVM model, the radial basis function (RBF kernel) is selected and the grid search method is used to optimize the SVM model parameters to achieve better performances. 

(2) Selecting samples that may be misclassified by SVM. According to the training model of SVM and the self-classification accuracy, selection conditions where the possibly misclassified samples belong to are determined. Analysis shows that samples distributed near the support vector or with the same number of votes during voting process are easily misclassified when SVM is used for classification. The SVM supports one-versus-one multiclassification. If *k* is the number of classes, *k(k-1)/2* models will be generated, each model involves only two class. Focusing on above problems, set the following conditions:

a) When using SVM for classification, if the highest number of votes are equal during the voting process, the sample will be regarded as a possibly misclassified sample and the sample will be picked out for referral.

b) If the test sample satisfies the following condition after input to SVM, this sample is selected for re-diagnosis.

(28) $$\left\{ \begin{array}{l} u_{\min}>v_{\min}\\ \frac{h_{\max}}{u_{\min}}>\frac{s_{\max}}{v_{\min}}\\ t_{1}<\frac{h_{\max}\cdot v_{\min}}{u_{\min}\cdot s_{\max}}<t_{2} \end{array}\right.,$$

where *u_min_* represents the smallest absolute value in the case of three votes; *h_max_* is the largest absolute value in the case of three votes; *v_min_* represents the smallest absolute value in the case of two votes; *s_max_* represents the largest absolute value in the case of two votes. The $t_{1}$ and $t_{2}$ are conditional parameters, which are determined by the performances of the trained SVM model, in this research, $t_{1}=1.5$ and $t_{2}=3.0$.

(3) Decision principles. When applying SVM-DT-ELM team-collaboration strategy for emotion identification, this paper follows the following principles:

ⅰ) If the test sample is classified by SVM with full votes and the sample is outside the set conditions, then DT and ELM are not employed for further consultation. The output emotional categories are based on the results of SVM.

ⅱ) Samples except those satisfying condition ⅰ) should be classified by DT and ELM. If any one of the results between DT and ELM are the same to SVM, the output emotional classes are based on the principle of minority obeying majority.

ⅲ) If the results of DT, ELM are different from the category of highest ranked vote of SVM and any one of the referral results between DT and ELM is consistent with the SVM’s second highest ranked vote, the final diagnosis category is based on the referral result.

(4) According to the principle of step (3), the emotional categories of the test samples are confirmed.

**Figure 4:**
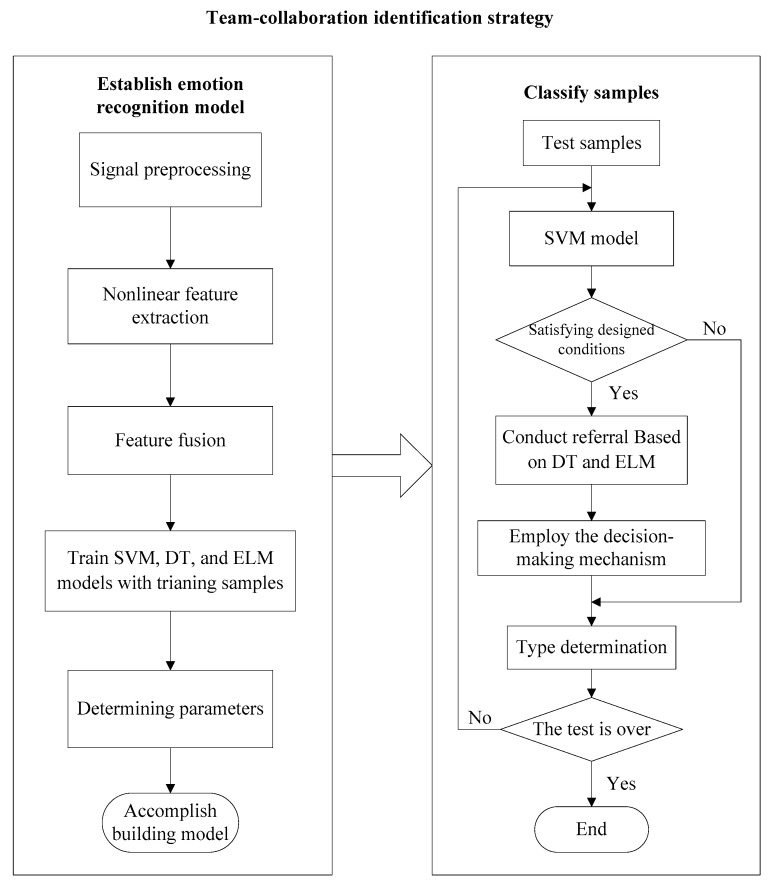
The flow chart of SVM-DT-ELM algorithm.

The flow chart of emotion recognition algorithm based on SVM-DT-ELM is shown in Figure 4.

## 3. Results and Discussions

The Augsburg Dataset and DEAP dataset were employed in order to fully verify the effectiveness of the proposed method. The performances of the proposed method were analyzed and compared with the exisiting studies from multiple perspectives, which highlights the significant ability of the proposed methods to recognize emotions through peripheral physiological signals.

### 3.1. Experiment Environment

All implementations are performed using MATLAB (R2015b) running on Windows 10 Laptop PC with Intel(R)Core (TM) i7-8750H CPU @ 2.21GHz processor with 16 GB RAM. Table 4 shows the hardware and software for the experiments.

### 3.2. Procedure of Emotion Recognition

**Figure 5:**
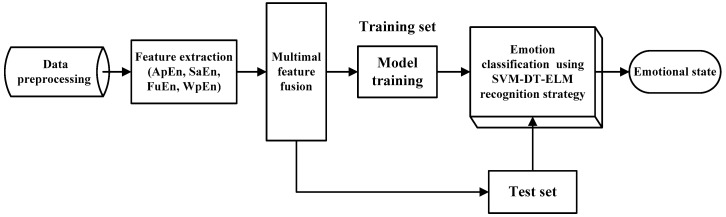
The architecture of emotion recognition with fused features and team-collaboration identification strategy.

Figure 5 illustrates the architecture of emotion recognition from physiological signals. Firstly, the raw emotional physiological signals need to be preprocessed. Then, the nonlinear features are extracted from four types of signals. Next, the extracted multimodal features are fused, the training samples are used to train the classifiers and test samples are classified with the proposed team-collaboration identification strategy.

### 3.3. Model Performance Evaluation Method

In order to quantify the performance of the proposed approach, the Hold-Out method is adopted to train and test the constructed classifier model, where the dataset is divided into two mutually exclusive sets, one is the training set and the other is the testing set. The 60%–80% of the dataset are usually randomly selected for training and the remaining are used for testing. Generally, the experiments need be repeated several times with random division and the average value is as the final result. Besides, the standard evaluation metric, accuracy (Acc), was used as a measurement to evaluate the performance of the above classification models and the values under different conditions are reported as mean ± standard. The calculation formula of average recognition rate and standard deviation is shown in (29) and (30):

(29) $$Acc^{*}=\frac{\sum_{i}^{N}Acc_{i}}{N}$$

(30) $$\sigma =\sqrt{\frac{\sum_{i}^{N}{\left(Acc^{*}-Acc_{i}\right)}^{2}}{N-1}},$$

where Acc_i_ represents the recognition accuracy of the i-th experiment, that is, the number of correctly classified samples divided by the total number of samples; N represents the number of experiments.

According to the size of the Augsburg dataset, 80% of the samples are used for training, while the remaining samples are used for testing. This procedure is repeated 10 times to ensure the results more reasonable. The accuracy values of 10 times was averaged as the final classification performance. When the experiments are conducted on the DEAP dataset, for each subject, 75% of the samples are used for training, while the remaining 25% are used for testing. Then, for each subject, the average classification performance is computed over the ten train-test repetition. 

### 3.4. Emotion Classification in Augsburg Dataset

In order to fully verify the effectiveness of the proposed method, the experimental results of emotion recognition are presented from three dimensions—(1) To show that the proposed feature level fusion is effective, the recognition results obtained by using features of a single signal are compared with that based on feature fusion; (2) To present the effectiveness of team-collaboration identification strategy, the recognition results obtained by using a single classifier to classify the samples with feature fusion are compared with the results based on the proposed team-collaboration identification strategy; (3) To further demonstrate the advantage of the proposed method, compare the results of this paper with that of related researches.

#### 3.4.1. Feature Level Fusion

Prior to classification of samples, four nonlinear features, ApEn, SaEn, FuEn and WpEn, were extracted from each physiological signal, including ECG, EMG, RSP and SC. Then the extracted features were fused and fed into classifiers for classification.

In order to show that the feature level fusion mechanism in this paper is effective, the recognition results of single physiological signal were compared with the recognition results of multimodal signals (the classifier uses the SVM, DT and ELM respectively). Table 5 shows the comparison of the 10-times average recognition rate of four types of emotional states between single signal features and feature fusion with multitype signals (Randomly selecting 500 samples each time). As can be seen the recognition accuracy can be effectively improved by the fusion at the feature level with each classifier. The high accuracy identification with fused features combining different classifiers clearly indicate that the features obtained from different signals containing complementary or supplementary information.

#### 3.4.2. Team-Collaboration Identification Strategy

In order to further improve the emotion recognition accuracy, the proposed SVM-DT-ELM team-collaboration identification strategy, is employed for classification. Firstly, 80 percent samples were randomly selected as training samples to establish the SVM, DT and ELM classification model respectively. According to the training model of SVM, selection conditions where the samples possibly misclassified were determined. The rest samples were used to test the performance of the proposed classification model and the experiments were repeated 10 times (Randomly selecting 500 samples each time). In addition, we also compared the classification accuracy of using SVM, DT and ELM with the recognition results of proposed method, as shown in Table 6.

Table 6 presents the accuracies of 10 experiments of using SVM, DT, ELM classifier and proposed team-collaboration identification strategy for four emotions when the features obtained from various physiological signals are taken in a fused manner. Compared with the accuracy of SVM classifier, the accuracy rate of the proposed SVM-DT-ELM reaches 98.6%, 3.1% higher than the result of SVM (95.5%). The proposed team-collaboration identification strategy presents better performances with 8.1% and 9.2% improved accuracy than DT and ELM, respectively. The experimental results demonstrate that the team-collaboration identification strategy is able to further improve the recognition accuracy and make the recognition results more reliable.

#### 3.4.3. Comparison with Existing Methods

Table 7 provides an overview of the accuracy rate comparisons of existing studies and the proposed method with Augsburg Dataset. Some studies for emotion recognition reported their performances based on valence and arousal measures only, which are not included here for comparison. As we can see, the recognition accuracy of this paper is increased to 98.6% with 16 features based on the proposed nonlinear features fusion and team-collaboration identification strategy. From the perspective of feature dimension and recognition accuracy, the extracted features have better performance than other features [29,51,52,53,54] and effectively reduce the feature dimension and improve emotion recognition accuracy. In addition, the proposed team-collaboration identification strategy can integrate the advantages of other classifications to effectively improve the classification accuracy.

### 3.5. Emotion Classification in DEAP Dataset

In this section, we present the results obtained from DEAP dataset to demonstrate the effectiveness of proposed methods. 

#### 3.5.1. Feature Level Fusion

In order to perform a more reliable classification process, we constructed a training set and a test set for each subject (s01, s02, s03, s04, s05). The number of training set is 300 and the test set is 100 for each subject. The experiments were carried out ten times and the accuracy rates of four dimensional emotions identification using single signals and multimodal signals with SVM, DT and ELM are shown in Table 8, Table 9 and Table 10, respectively. 

It can be seen in Table 8, Table 9 and Table 10 that the classification performances of RSP and EMG are good on the whole, presenting their certain advantages on detecting different emotions. These two physiological signals also perform well in Augsburg dataset, which is where the two databases are consistent. Compared with the other three signals, the classification accuracy of GSR is not good all in all. The results of BVP is more satisfying. In addition, it can be seen that the results of multi sensors fusion present much improvement comparing with the results of single signal for each subject. Performance analysis shows that multi-type information fusion can effectively improve the accuracy of emotion identification, which is consistent with the experimental results with the Augsburg dataset.

#### 3.5.2. Team-Collaboration Identification Strategy

**Figure 6:**
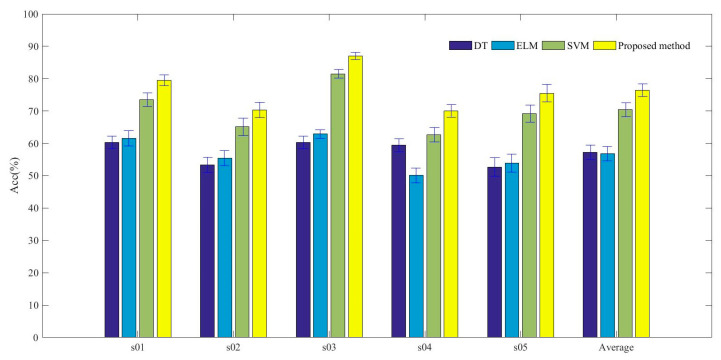
Average accuracy of each subject using SVM, DT, ELM and proposed method for four types of emotional identification.

In this research, the team-collaboration identification strategy is proposed to avoid the limitations of a single classification method. The results of ten times experiments are shown in Table 11, comparing between SVM, DT, ELM and the proposed method for the five subjects individually. Due to individual differences, the accuracy of emotion recognition varies among different subjects. However, as can be seen, the results illustrate the improvement in the classification accuracy for each subject after using the proposed classification model. Taking the subject s01 as an example, when employing the proposed strategy, the average accuracy improved by 6% than the SVM classfication method, from 73.5% to 79.5%. And there were 19.2% and 18% improved accuracy than the rusults of DT and ELM, respectively. Overall, the average identification accuracies are 70.4% and 76.46% for SVM and proposed strategy, respectively, improving with 6.06%. Meanwhile, when the proposed method was employed, there were smaller mean square deviation, which present the better stability of the team-collaboration identification strategy than the other methds. The compared results for each subject with SVM, DT, ELM and proposed method are demonstrated in Figure 6. 

#### 3.5.3. Comparison with Existing Methods

In recent years, various studies of emotion recognition have been conducted on DEAP dataset. Generally, The research work mainly focuses on two dimensional classification (HA/LA, HV/LV) or four dimensional classification (HVHA, HVLA, LVLA, LVHA).

The previous studies [55,56,57,58,59] based on two dimensional emotion classes using DEAP dataset are shown in Table 12. As can be seen from Table 12, the methods adopted [47,48,49,50,51] are able to identify the two types of emotions to a certain extent. However, binary emotion model is difficult to fully express emotional states. Therefore, this paper takes four dimensional emotion classes (HVHA, HVLA, LVLA, LVHA) into consideration.

In this investigation, the emotional states were identified from subject-dependent perspective. The training and testing were performed on the same subject, which was same as the Ref [58,60,61]. An overview comparison of accuracy rate based on four dimensional emotion classes with different methods is shown in Table 13. In this paper, the identification accuracy of the four types of emotions was 76.46%. It can been seen that the proposed method with fused nonlinear features and team-collaboration identification strategy presents better identification performance than the existing methods in References [58,60,61]. Although only five experimental subjecs (s01~s05) were employed for analysis in this paper, the analysis results are not affected, because the subjects were selected sequentially, not deliberately. In addition, the studies of References [58,60,61] have been carried out based on EEG signals, while this paper is based on physiological signals. The experimental results show that as long as the method is proper, the use of physiological signals can also achieve good emotion recognition. In this investigation, the effectiveness of proposed methods of feature representation and classification were verified by comprehensive analysis with Augsburg dataset and DEAP dataset. The experimental analysis results of two datasets are consistant that the proposed methods present good performances of emotion identification, which indicates the universality of the proposed methods. 

### 3.6. Discussions

In this investigation, the method of nonlinear features extraction and multi-signal feature fusion was proposed to effectively characterize emotional responses and team-collaboration identification strategy was suggested to improve the accuracy of emotion recognition. The effectiveness of the proposed feature extraction fusion method and classification method were verified by single factor analysis comparision on Augsburg and DEAP datasets respectively. The results before and after feature fusion were compared to verify the effectiveness of the proposed feature fusion method. The effectiveness of the proposed classification method was verified by comparing the results of the single classification method with that of the team-collaboration identification strategy. Meanwhile, the validity of the proposed methods were fully verified by comparing with the results of existing research methods based on the same database. Whether it was the comparison of the effect before and after the fusion or the comparison with the methods of other researchers, the methods proposed in this article have shown superior performances. 

Various methods of feature extraction and classification [51,52,53,54,55,56,57,58,59,60,61] were proposed and employed by researchers, in terms of using physiological signals to identify emotions. No matter which method was adopted, the purpose was to improve the accuracy of emotion identification. Recently, deep learning methodologies have become popular to analyse physiological signals and employ to realize emotion classification [5]. Kwon et al [59] employed deep learning method to conduct two dimensional emotion identification on DEAP dataset. At present, there is no application of deep learning methods for the same research goal as this article on the Augsburg and DEAP datasets. Hence, the results of this paper were not compared with that of deep learning method. In this research, at present, we have studied the emotion recognition of a single person using physiological signals. In the future work, further investigation is needed on how to extract more discriminative features to make cross-subject emotion classification and how to construct and optimize classification models with higher accuracy for emotion recognition and how to effectively adopt deep learning method to identify emotions with peripheral neurophysiological signals and EEG signals.

## 4. Conclusions

In this paper, the methods of emotional state identification based on physiological signals were investigated. In order to represent emotional states effectively, the method of nonlinear features extraction and multi-signal feature fusion was suggested. Meanwhile, team-collaboration identification strategy was proposed for avoiding the limitations of a single classifier. The four nonlinear features, namely ApEn, SaEn, FuEn and WpEn were employed and extracted from each physiological signal, then the extracted features of different physiological signals were fused, for example fusion of ECG, EMG, RSP, SC with Augsburg dataset and EMG, RSP, BVP, GSR with DEAP dataset. Nonlinear features can represent emotion characteristics in depth and multi-signal feature fusion can represent emotion from multiple perspectives. Hence, the proposed feature represent method is more effective. In order to improve the accuracy of emotion identification, team-collaboration identification strategy was proposed. According to the performances and characteristics of SVM, DT and ELM classifiers, the team-collaboration decision-making mechanism was developed, which according to the difficulty of sample classification, single classification decision and team-collaboration identification can be adopted respectively. The proposed strategy can choose the appropriate decision method according to the characteristics of the samples and can effectively integrate the advantages of other classifiers to avoid the limitations of single classifier. The Augsburg dataset and DEAP dataset were employed to verify the validity and universality of the proposed method. The experimental results from the two public databases uniformly indicated that the proposed method combining fused nonlinear features and team-collaboration identification strategy has better performances than the existing methods. 

## Figures and Tables

**Table 1:** The content summary of Augsburg Database.

Elicitation Material	Music
Emotional states	Joy, anger, sadness, pleasure
Number of subjects	1
Collected signals	ECG, EMG, RSP, SC
Length	120 seconds
Sampling frequency	ECG: 256 Hz; EMG, RSP and SC: 32 Hz;
Collected days	25

**Table 2:** The preprocessed DEAP database content summary.

Elicitation Material	Videos
Emotion labels	Arousal, valence
Number of subjects	32
Collected signals	EEG, EOG, GSR, BVP, RSP, EMG, SKT
Length	60 seconds
Sampling frequency	128 Hz;
Rating values	Arousal: 1–9 Valence: 1–9

**Table 3:** The number of samples for four dimensional emotional states of each subject.

Subject	HVHA	HVLA	LVLA	LVHA	Total
s01	130	60	100	110	400
s02	160	60	100	80	400
s03	10	210	110	70	400
s04	120	40	200	40	400
s05	130	110	100	60	400
Total	550	480	610	360	2000

**Table 4:** The hardware and framework specifications.

CPU	Intel Core i7-8750H
GPU	NVIDIA GeForce GTX1050Ti 4GB
OS	Windows 10
RAM	DDR4 16GB
Frameworks	MATLAB (R2015b)

**Table 5:** Comparison of the performances between single signal features and feature fusion of multitype signals.

	Physiological Sensor	Acc^*^(%)		
		SVM	DT	ELM
Single sensor	ECG	65.7 ± 1.55	62.1 ± 1.92	58.4 ± 2.72
	EMG	72.1 ± 0.97	60.1 ± 1.84	62.3 ± 2.47
	RSP	66.4 ± 1.22	66.2 ± 1.75	59.7 ± 2.62
	SC	70.9 ± 1.43	64.5 ± 1.54	60.6 ± 2.33
Multi sensors	ECG + EMG + RSP + SC	95.5 ± 0.85	90.5 ± 1.27	89.4 ± 1.78

**Table 6:** An overview of the comparison of the classification accuracy using different classifiers.

Number of Experiments	Classification Methods (Acc^*^/%)			
	SVM	DT	ELM	SVM-DT-ELM
1	96	91	86	98
2	96	91	89	98
3	95	92	89	98
4	95	89	91	99
5	94	90	88	98
6	95	90	90	98
7	95	93	92	99
8	96	89	90	100
9	96	90	88	99
10	97	90	91	99
Acc^*^ (%)	95.5 ± 0.85	90.5 ± 1.27	89.4 ± 1.78	98.6 ± 0.70

**Table 7:** Accuracy comparison of various studies.

Classification Method	Feature Dimension	Acc^*^ (%)
LDF [29]	32	92.05
SVM [51]	64	95
PSO-SNC [52]	32	86
SVM [53]	28	76
C4.5 DT [54]	155	93
This paper	16	98.6

**Table 8:** The classification accuracy for each subject using single signal and multimodal signals with SVM.

Subject	Physiological Sensors				
	Single Sensor (Acc^*^/%)				Multi Sensors (Acc^*^/%)
	GSR	RSP	BVP	EMG	GSR + RSP + EMG + BVP
s01	38.0 ± 1.46	43.3 ± 2.73	53.5 ± 3.06	50.6 ± 1.56	73.5 ± 2.07
s02	34.0 ± 2.25	53.1 ± 1.73	43.2 ± 1.67	52.1 ± 3.03	65.1 ± 2.69
s03	54.3 ± 1.51	60.8 ± 2.72	65.6 ± 1.83	63.2 ± 2.33	81.5 ± 1.35
s04	48.6 ± 4.13	52.3 ± 3.67	56.5 ± 3.99	56.1 ± 2.17	62.7 ± 2.21
s05	32.8 ± 2.56	43.1 ± 3.88	47.6 ± 3.69	42.5 ± 3.04	69.2 ± 2.70

**Table 9:** The classification accuracy for each subject using single signal and multimodal signals with DT.

Subject	Physiological Sensors				
	Single Sensor (Acc^*^/%)				Multi Sensors (Acc^*^/%)
	GSR	RSP	BVP	EMG	GSR + RSP + EMG + BVP
s01	32.8 ± 2.95	39.8 ± 3.11	41.6 ± 4.34	46.4 ± 2.41	60.3 ± 1.95
s02	35.6 ± 1.82	33.0 ± 2.23	50.0 ± 2.35	44.2 ± 3.70	53.3 ± 2.36
s03	40.8 ± 1.30	49.4 ± 2.70	55.2 ± 0.84	57.4 ± 1.82	60.3 ± 2.00
s04	40.8 ± 2.17	40.2 ± 3.03	40.4 ± 4.16	45.4 ± 1.95	59.4 ± 2.01
s05	28.6 ± 1.52	41.4 ± 2.40	37.2 ± 1.48	41.6 ± 2.30	52.7 ± 2.83

**Table 10:** The classification accuracy for each subject using single signal and multimodal signals with ELM.

Subject	Physiological Sensors				
	Single Sensor (Acc^*^/%)				Multi Sensors (Acc^*^/%)
	GSR	RSP	BVP	EMG	GSR + RSP + EMG + BVP
s01	29.2 ± 1.64	47.0 ± 2.55	46.8 ± 1.92	43.4 ± 3.65	61.5 ± 2.37
s02	30.4 ± 1.67	34.4 ± 2.70	49.6 ± 3.50	42.6 ± 1.82	55.4 ± 2.37
s03	40.0 ± 3.08	54.6 ± 3.36	54.4 ± 2.97	54.6 ± 2.30	62.9 ± 1.29
s04	39.6 ± 2.70	47.8 ± 2.56	42.6 ± 3.21	45.8 ± 3.56	50.1 ± 2.28
s05	34.8 ± 3.35	44.4 ± 2.97	39.8 ± 3.83	43.2 ± 3.11	53.9 ± 2.73

**Table 11:** Comparison of the results using SVM, DT, ELM and proposed classification strategy for each subject.

Subject	Method	The Identification Accuracy of Each Experiment (%)										Average (%)
		1	2	3	4	5	6	7	8	9	10	
s01	DT	64	61	58	60	58	59	59	60	62	62	60.3 ± 1.95
	ELM	62	60	64	58	62	58	64	60	64	63	61.5 ± 2.37
	SVM	75	72	72	76	75	77	72	73	71	72	73.5 ± 2.07
	Proposed	80	80	80	81	80	82	79	79	76	78	79.5 ± 1.65
s02	DT	55	51	53	53	50	56	51	57	52	55	53.3 ± 2.36
	ELM	58	54	59	52	53	56	53	57	57	55	55.4 ± 2.37
	SVM	68	66	71	63	63	64	64	66	63	63	65.1 ± 2.69
	Proposed	72	70	76	70	69	70	68	70	68	70	70.3 ± 2.31
s03	DT	62	58	59	63	62	61	57	59	60	62	60.3 ± 2.00
	ELM	65	63	61	63	62	62	63	63	65	62	62.9 ± 1.29
	SVM	84	82	80	82	83	81	80	81	80	82	81.5 ± 1.35
	Proposed	88	88	87	89	86	86	87	86	87	86	87 ± 1.05
s04	DT	58	62	60	57	61	58	59	58	63	58	59.4 ± 2.01
	ELM	48	51	49	47	53	50	47	52	53	51	50.1 ± 2.28
	SVM	63	66	65	64	65	60	61	60	62	61	62.7 ± 2.21
	Proposed	70	70	72	72	73	68	70	70	68	67	70 ± 1.94
s05	DT	50	52	52	55	50	51	59	51	52	55	52.7 ± 2.83
	ELM	57	58	52	50	56	53	54	51	52	56	53.9 ± 2.73
	SVM	69	67	68	66	76	69	70	70	69	68	69.2 ± 2.70
	Proposed	75	73	78	73	82	75	75	74	75	75	75.5 ± 2.68
Overall average	DT ELM SVM											57.2 ± 2.23 56.8 ± 2.21 70.4 ± 2.20
	Proposed											76.46 ± 1.93

**Table 12:** Accuracy comparison of various studies in two-dimensional classification.

Method	Acc^*^ (%)	
	Arousal	Valence
Chen et al. [55]	69.09	67.89
Zhuang et al. [56]	71.9	69.1
Yin et al. [57]	77.1	76.1
Alazrai et al. [58]	86.6	85.8
Kwon et al [59]	76.56	80.46

**Table 13:** Accuracy comparison of various studies.

Menthod	Emotions	Acc^*^(%)
M Zubair and C Yoon [60]	HVHA, HVLA, LVLA, LVHA	49.7
Alazrai et al [58]	HVHA, HVLA, LVLA, LVHA	75.1
Zheng et al [61]	HVHA, HVLA, LVLA, LVHA	69.67
This paper	HVHA, HVLA, LVLA, LVHA	76.46
